# Glucose Homeostasis Improvement After Single Anastomosis Duodenojejunal Bypass with Sleeve Gastrectomy in Goto-Kakizaki Rats

**DOI:** 10.1007/s11695-025-07799-4

**Published:** 2025-03-25

**Authors:** Sirio Melone, Jose Maria Fernandez-Cebrian, Mario Amores, Yolanda Lopez-Tofiño, Elia Perez-Fernandez, Elena Garcia-Garcia, Juan Manuel Acedo, Carlos Guijarro, Sagrario Martinez Cortijo, Raquel Abalo, Maria Ruth Pazos

**Affiliations:** 1 Universidad Rey Juan Carlos, Alcorcón, Spain; 2 Hospital Universitario Fundación Alcorcón, Madrid, Spain; 3 Hospital Universitario Ramón y Cajal, Madrid, Spain; 4 Universidad Francisco de Vitoria, Pozuelo de Alarcón, Spain

**Keywords:** Metabolic bariatric surgery, Duodenojejunal bypass, Diabetes mellitus, Goto-Kakizaki rats

## Abstract

**Background:**

The incidence of type 2 diabetes mellitus (T2DM) is raising with significant associated medical complications and mortality. Bariatric surgery has shown to have beneficial metabolic effects. A model of single anastomosis duodenojejunal bypass with sleeve gastrectomy (SADJB-SG) was developed in a T2DM animal model without obesity, Goto-Kakizaki (GK) rats, to evaluate the effect of the procedure on glucose homeostasis.

**Methods:**

Fourteen 12-week old GK rats underwent SADJB-SG, while 11 underwent simulated surgery (Sham). Weight and food intake were recorded comprehensively until sacrifice. Fasting blood glucose data, as well as insulin, fructosamine, and albumin levels were measured both pre-surgically and just before sacrifice. Glucose homeostasis was also monitored by oral glucose tolerance test (OGTT) at different time points. A radiographic study was performed to assess the effect of surgery on gastric emptying.

**Results:**

Mortality rate was 24% in the SADJB-SG and 4% in Sham rats. Despite similar food intake, the SADJB-SG showed significant weight loss coupled to a decrease in albumin levels. Glucose homeostasis improved in SADJB-SG rats after surgery, reflected in decreased blood glucose, fructosamine levels, and homeostasis model assessment of insulin resistance index (HOMA-IR). OGTT tests, conducted both post-surgery and at follow-up, demonstrated an improvement in glucose metabolism 120 min after glucose administration. However, a peak in glycemia was observed at 30 min, which negatively affected the expected *AUC* results. Gastric emptying was accelerated in the SADJB-SG, which could contribute to explain the observed glycemia increment, through fast glucose jejunal uptake.

**Conclusion:**

SADJB-SG surgery improved glucose homeostasis in GK rats.

## Introduction

Type 2 diabetes mellitus (T2DM) is a very complex and heterogeneous metabolic disorder associated with a significant risk for the development of different pathologies, such as cardiovascular disease, stroke, retinopathy, renal disease, dementia, and even several types of cancer [1]. Indeed, the impact of T2DM on associated medical problems, mortality, quality of life, and healthcare costs have been well described elsewhere [1–4]. Based on the study published by the International Diabetes Federation, there were an estimated 463 million people with DM in the world in 2019, and the number of people affected by the disease is expected to increase to 578 million by 2030 and to 700 by 2045 [2, 5, 6]. The increase in the incidence of DM appears to be primarily due to the increment in type 2 DM (T2DM) [3]. The close relationship between T2DM and obesity has been known since the early twentieth century, so that an elevated body mass index (BMI) is considered a risk factor for developing T2DM [7]. The mechanisms involved are not yet properly elucidated, although obesity can be linked to the insulin resistance associated with T2DM. Furthermore, insulin resistance could be due to activation of inflammatory cascades, mitochondrial dysfunction, or hyperinsulinemia associated with obesity [8].

Potential beneficial metabolic effects of weight reduction surgeries have been demonstrated [9], in particular procedures that surgically bypass the small bowel, such as Roux-en-Y gastric bypass (RYGB) and duodenal switch (DS). Furthermore, in many cases, this improvement in glycemic control occurs even before significant weight loss after surgery, raising the hypothesis that there are weight loss independent mechanisms involved in DM amelioration [10, 11].

Single anastomosis duodenojejunal bypass with sleeve gastrectomy (SADJB-SG) was first reported in humans by Lee and colleagues [12]. This procedure combines the knowledge of Kasama’s sleeve gastrectomy with duodenojejunal exclusion performing a Roux-en-Y reconstruction [13], and Sanchez-Pernaute’s single anastomosis approach [14]. SADJB-SG achieved significant and sustained weight loss, as well as T2DM remission rates similar to those of RYGB [15]. The main advantage of this procedure is that performing a single anastomosis reduces the operative time as well as surgical complications. Additionally, providing a shorter loop in SADJB-SG could prevent malnutrition, commonly associated with malabsorptive techniques, such as DS, biliopancreatic diversion (BPD), or single anastomosis duodenoileal bypass with sleeve gastrectomy (SADI-S) [15].

Recently, we developed the SADJB-SG technique in a murine model of T2DM, the Goto-Kakizaki (GK) rat [16]. Unlike other animal models of T2DM, GK rats are not animals with obesity, which provides a suitable model for assessing the effects of SADJB-SG on glucose metabolism independently of the effect of obesity [17]. Therefore, the aim of the present study was to evaluate the effect of SADJB-SG technique on glycemic regulation in this animal model.

## Materials and Methods

### Animals

All the animal experimental procedures were approved by the Ethical Committee for Animal Welfare of the Universidad Rey Juan Carlos and Comunidad Autónoma de Madrid, Spain (PROEX 281/19). The experimental protocol met European and Spanish (2010/63/EEC and RD 53/2013) guidelines for the protection of experimental animals. Nine-week-old GK rats purchased from the Miguel Hernández University (Alicante, Spain) were used. Following the quarantine period, the animals were housed and bred individually in environmentally controlled conditions (22 ± 1 °C of temperature, 60% of humidity, and 12 h light/dark cycle), and had access to filtered and sterilized tap water and food (1320 formula; Altromin International, Germany) ad libitum. Wood-based aspen bricks were used for bedding (Sodispan Biotech, Spain). Besides, rats were provided with environmental enrichment items for nest building, chewing, and hiding (Sodispan Biotech, Spain). During the fasting periods, the environmental enrichment elements were removed.

**Fig. 1:**
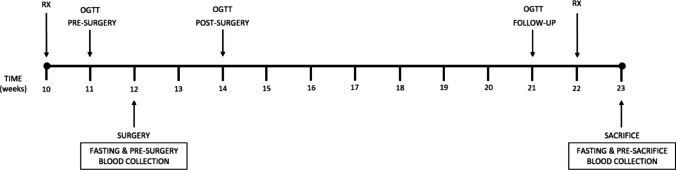
Experimental timeline. The animals were subjected to a weekly control to evaluate their evolution, weight, and food intake. This control was particularly exhaustive during the first postoperative week to identify and mitigate possible painful symptoms and dehydration. RX, Radiological study; OGTT, oral glucose tolerance test

The animals were subjected to an exhaustive pre- and post-operative control, as previously published [16]. Due to sex-based differences in T2DM and, in particular, in GK rats, only male rats were used to avoid variability in our results mainly due to the influence of estrogens in females [18]. Two experimental groups were established: (1) animals subjected to surgery (SADJB-SG) and (2) animals subjected to simulated surgery (SHAM). The animals were randomly distributed between the two groups including 11 rats in the SHAM group and 14 in the SADJB-SG group. The timeline and general scheme of the procedures performed are shown in Fig. 1.

### Surgical Procedure

Based on the results of previous work, SADJB-SG was performed using the duodenal transection technique for duodenal exclusion in 12-week-old GK rats [16]. Briefly, after 6 h of fasting, the rats were anesthetized, and a 3-cm midline abdominal incision was made. Then, the ligament of Treitz was identified and a side-to-side duodenojejunal anastomosis was performed 3–5 mm distal to the pylorus. Subsequently, the first portion of the duodenum was excluded by transection, just distal to the anastomosis. Finally, a tubular gastrectomy of approximately 70% of the stomach was performed. Sham surgery consisted in laparotomy, gastrotomy, and gastrorrhaphy. The animals were subjected to a weekly control to assess their evolution, except during the first postoperative week, when the evolution of the animals was monitored daily.

### Body Weight and Food Intake Record

Animal body weight was recorded weekly from 2 weeks before surgery until sacrifice. Food intake was also monitored weekly, except during the 7 days after surgery when the animals received palatable gelatin tablets, prepared as previously described [16], until their correct evolution was verified. From the third postsurgery day, the solid diet was gradually introduced according to their tolerance. From the second week post-surgery, all animals received a solid diet, and the weekly control was reinitiated.

### Fasting Glucose and OGTT

Before blood sampling, the animals were subjected to 6 h of fasting. While fasting, rats had free access to water. Glycemia was measured using a glucometer and reactive strips (Accu-Chek® Performa; Roche, Spain). Blood drops are obtained from the tail in conscious rats at different time points (Fig. 1).

Oral glucose tolerance test (OGTT) was conducted before surgery (week 11) and after surgery, specifically at week 14 (post-surgery) and 21 (follow-up). Fasting glycemia was measured and then a glucose solution (2 mg/kg; Glucocemin 50%, Braun) was administered orally. Thirty, 60, and 120 min after glucose administration, glycemia was again measured. Then, the area under the curve (*AUC*) was calculated from OGTT data [19].

### Insulin, Fructosamine, and Albumin Measurements

Different parameters related to glucose metabolism or nutritional condition of the animals was measured just before surgery and before sacrifice. After 6 h of fasting, blood samples were collected from the tail vein in pre-chilled tubes containing EDTA for insulin preservation. A protease inhibitor (dipeptidyl dipeptidase inhibitor IV; Merck-Millipore, Spain) was immediately added following blood extraction. The samples were centrifuged at 3000 rpm and 4 °C, for 10 min, and plasma was collected and frozen at − 80 °C. Fructosamine and albumin levels were determined on the Abbott Alinity analyzer (Abbott; Illinois, USA). An enzyme-linked immunosorbent assay (ELISA) kit was used to measure blood insulin concentration (Cloud-Clone Corp; USA) following the manufacturer’s guidelines. Fasting plasma glucose and insulin levels were used to calculate homeostasis model assessment of insulin resistance (HOMA-IR) [20].

### Radiographic Study

To analyze the effect of surgery on the stomach size, as well as to evaluate a possible alteration in gastric emptying, a radiographic study was carried out at week 11 (pre-surgery) and 22 (post-surgery), as previously described [21], using the intragastric administration of 1.5 mL of barium sulfate (Barigraph® AD, Juste SAQF, Spain; 2 g/ml). Image acquisition was performed at different time points after barium administration: immediately (0) and 1, 2, 3, 4, 6, 8, and 24 h later. Radiographs were taken using a CS2100 digital X-ray device (Carestream Dental; 60 kV, 7 mA) and recorded on a digital plate (Venu1717V, iRay Technologies). The rats did not receive anesthesia and were immobilized in a prone position inside transparent, handmade, and adjustable plastic tubes. Exposure time was set to 20 ms (focus distance 50 ± 1 cm), and the rats were returned to their cage immediately after each X-ray shot. A metal block (3 × 1 × 1 cm) was placed aside the animal during X-ray obtention and used as reference for morphometric and densitometric analyses. For these, the size (measured as area) as well as the density of the stomach contents (measured as %) were analyzed using an image analysis system (ImageJ 1.38, National Institute of Health, USA).

### Histological Study

Animals were deeply anesthetized with isoflurane and sacrificed by decapitation. Tissue samples were dissected and fixed in 4% formaldehyde for 24 h before embedding in paraffin. Esophageal samples were cut on a Leyca microtome (Leyca; Wetzlar, Germany) to obtain 4-μm-thick sections. Sections were stained with hematoxylin–eosin for histological evaluation by gastrointestinal pathologist, blinded to the animal group. Representative light microphotographs were acquired with a DMD108 photomicroscope (Leyca; Wetzlar, Germany).

### Statistical Analysis

STATA 17 statistical software (StataCorp.2021. Stata Statistical Software: Release 17. StataCorp LLC, USA) was used to perform statistical analyses. Linear mixed models were conducted to analyze longitudinal measures, with an unstructured variance matrix to account for variability. The models included treatment group (Sham or SADJB-SG) as fixed effect, time as repeated measure, and first level interaction time × treatment. *Mean* and standard error (*SE*) were estimated, and pairwise comparisons were adjusted using the Bonferroni method. Statistical significance was defined as *p < 0.05 and **p < 0.001.

## Results

### Surgery Evaluation

All interventions were successfully performed on 25 rats. The mean operative time was 29 and 78 min for the Sham and SADJB-SG groups, respectively. Anesthetic time was deliberately prolonged in the Sham group to avoid bias regarding surgical stress and anesthesia effects. Overall, mortality rate was 28%; however, 24% corresponded to the SADJB-SG group, since only one rat died in the Sham group (4%), due to duodenal ischemia. In SADJB-SG group, the most common cause of death was anastomosis leakage in 3 cases, gastric leak in one case, and duodenal ischemia in one case.

### Body Weight, Food Intake, and Nutrition Parameters

**Fig. 2:**
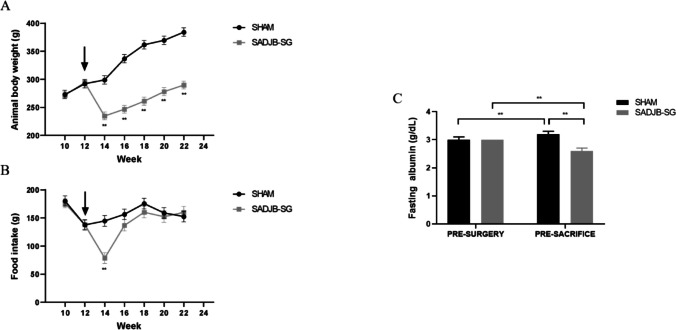
Body weight (**a**) and food intake (**b**) were recorded weekly. Arrowheads indicate the surgical procedure. **c** Serum albumin levels were measured on surgery day and before sacrifice. Linear mixed models were used for repeated measures analysis over time. Estimated mean ± *SE* (standard error) was represented, and Bonferroni method was used to calculate adjusted *p*-values: **p < 0.001

As shown in Fig. 2 a, after surgery, the SADJB-SG group experiences substantial weight loss (average of 21%). Although operated rats gradually gained weight, significant differences compared to the Sham group were maintained over the time (*p* < 0.001). The Sham group quickly recovered their initial baseline body weight, which continued to increase progressively. Regarding food intake, SADJB-SG rats showed a decrease between weeks 12 and 14, just after surgery, as expected due to the post-surgical recovery period (Fig. 2b). Following recovery and introduction of solid diet (weeks 14–16), there was an increase in the food intake in the operated rats reaching similar values as the Sham group until the end of the study.

Serum albumin levels in Sham rats revealed a significant increment at the time of sacrifice compared to basal values (Fig. 2c). On the opposite, SADJB-SG group evidenced a decrease in albumin levels at the time of sacrifice when compared to the basal measurement (*p* < 0.001). Moreover, SADJB-SG rats had lower albumin blood concentration in the sample obtained at sacrifice compared to the Sham group (*p* < 0.001), consistent with body weight data.

#### OGTT

**Fig. 3:**
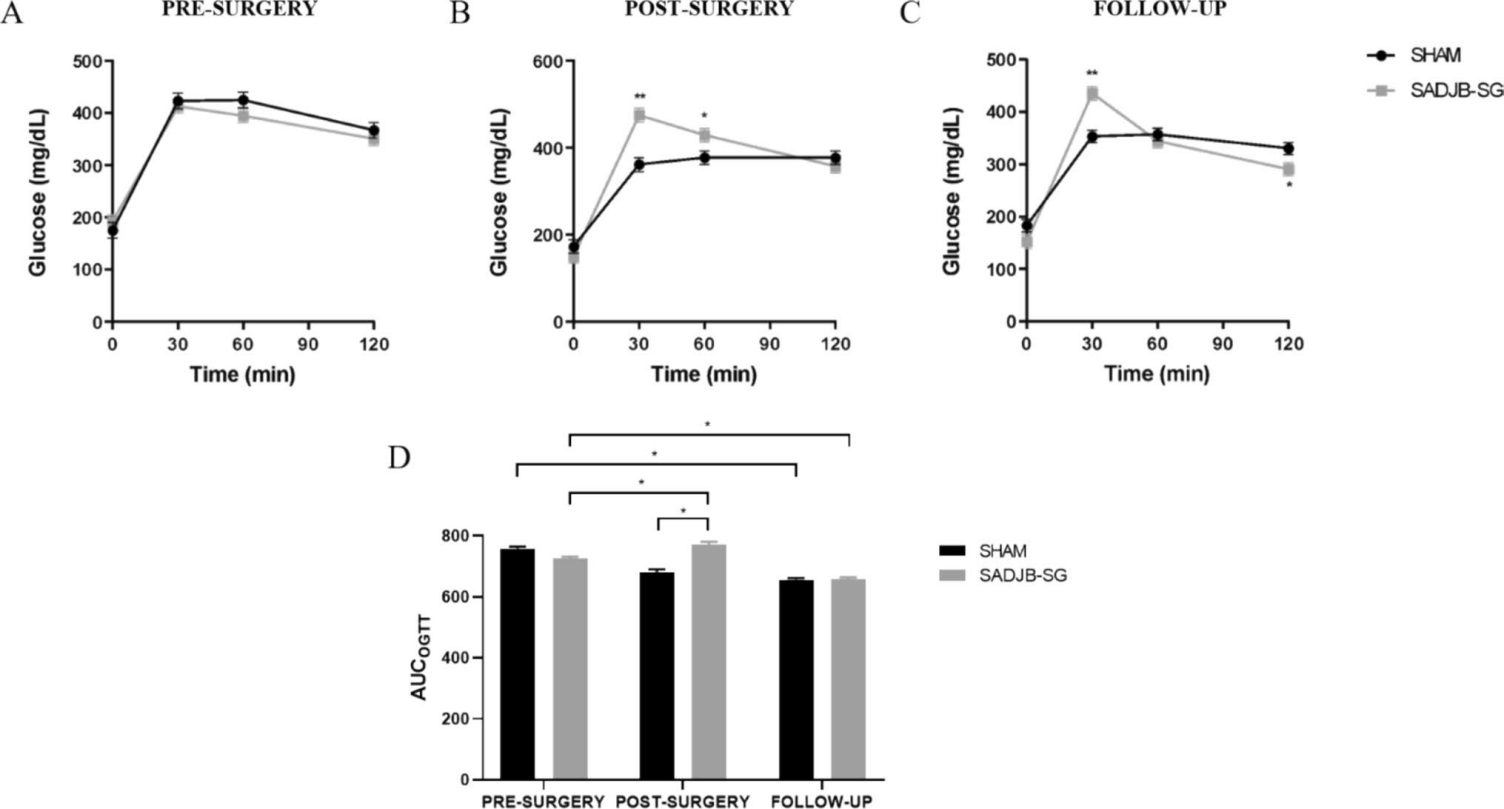
The OGTT was performed prior to surgery (**a**), 2 weeks (**b**), and 9 weeks (**c**) post-surgery. **d** The OGTT data were used to calculate the area under the curve (*AUC*). Linear mixed models were used for repeated measures analysis over time. Estimated *mean* ± *SE* (standard error) was represented, and Bonferroni method was used to calculate adjusted *p*-values: *p < 0.05 and **p < 0.001

The OGTT performed before surgery showed a similar profile in both experimental groups (Fig. 3a). In post-surgery (Fig. 3b) and follow-up (Fig. 3c) tests, there was an important increase in blood glucose concentration 30 min after glucose overload in SADJB-SG (*p* < 0.001). Following glycemic increment, the glucose concentration declined to levels similar to those of the Sham group at 120 min. Moreover, in the follow-up test, glycemia in the SADJB-SG group was significantly lower than in Sham at 120 min (Fig. 3c). Calculated *AUC*_OGTT_ was higher in SADJB-SG rats (*p* < 0.05) compared to Sham group in the OGTT test conducted post-surgically (Fig. 3d). However, the *AUC*_OGTT_ calculated in the follow-up period, decreased in both experimental groups compared to the pre-surgery values (*p* < 0.05).

### Glucose Metabolism Indicators

**Fig. 4:**
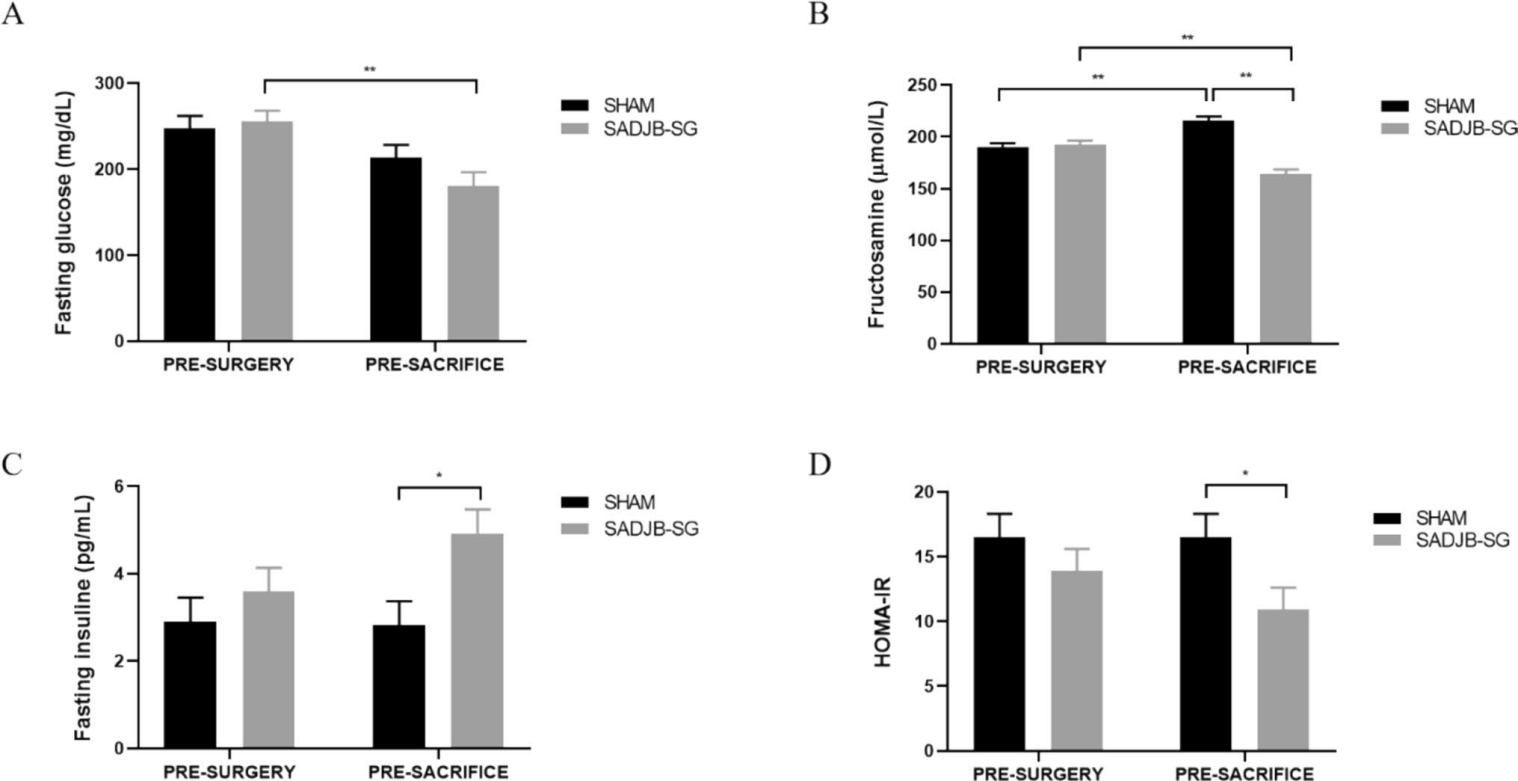
Graphical representation of the glycemia-related parameters. **a** Fasting glucose, **b** fructosamine, and **c** insulin blood concentrations were measured just before surgery and sacrifice. **d** Homeostasis model assessment of insulin resistance (HOMA-IR). Data represent estimated *mean* ± *SE* (standard error), and Bonferroni method was used to calculate adjusted *p*-values: *p < 0.05 and **p < 0.001

Fasting glucose levels in SADJB-SG animals were lower 12 weeks after surgery (Fig. 4a). In addition, it was also observed that in the Sham group, fructosamine levels at sacrifice were increased compared to the basal measurement, whereas the surgery significantly reduced (*p* < 0.001) long-term fructosamine levels in SADJB-SG (Fig. 4b). Regarding fasting insulin levels, a significant increase (*p* < 0.05) was observed in SADJB-SG group at sacrifice (Fig. 4c). On the other hand, HOMA-IR was lower in the SADJB-SG group (Fig. 4d), indicating a reduced insulin resistance in these animals.

## Effects of SADJB-SG Surgery on Stomach Size and Gastric Emptying

**Fig. 5:**
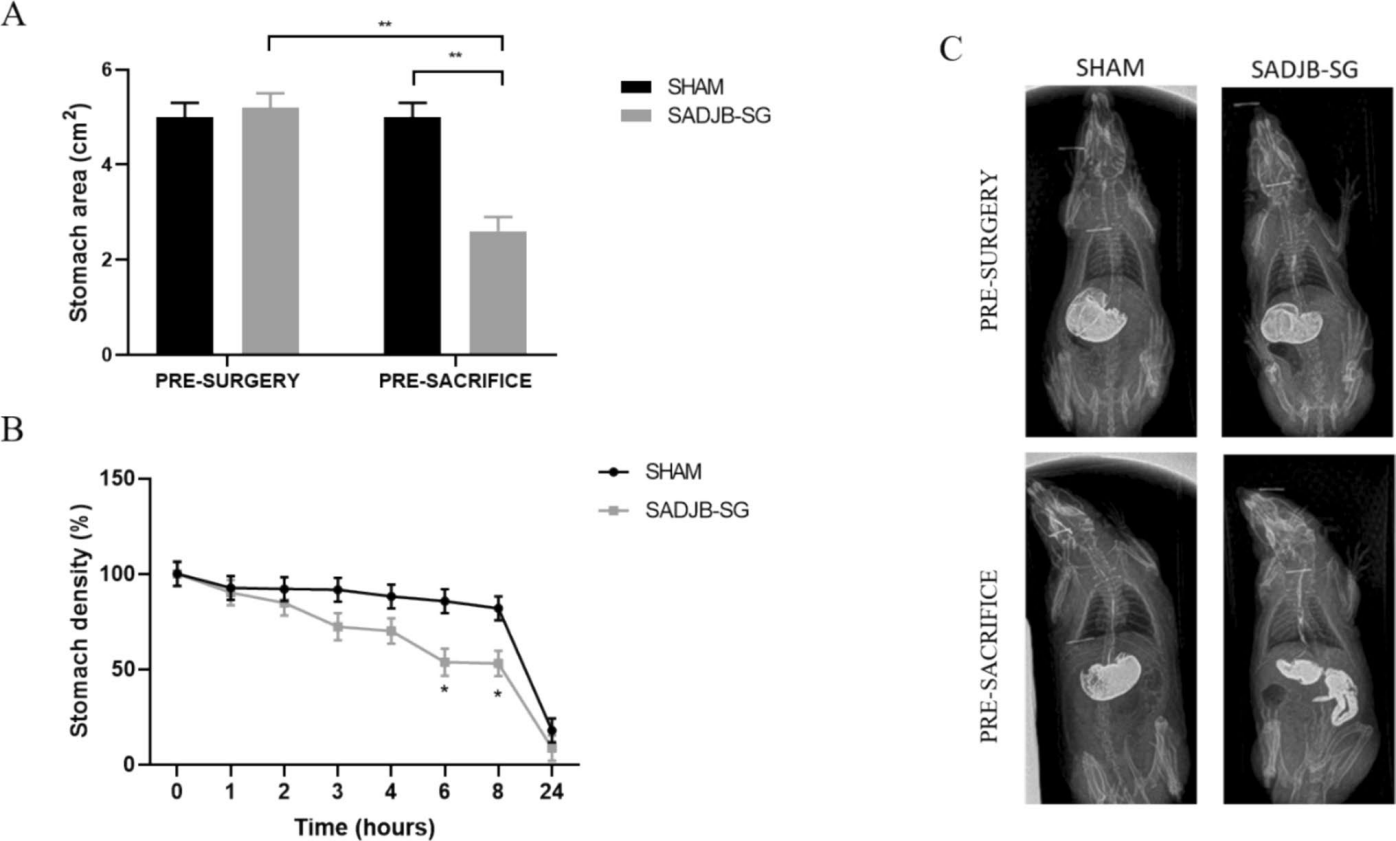
Radiological study and analysis of the images using the ImageJ software. **a** Stomach area analysis was performed at T0, right after barium administration, both pre-surgically and pre-sacrifice. The reduction observed after surgery was 42% in the SADJB-SG group. **b** Analysis of the contrast density in the stomach for 24 h after barium administration showed an increase in gastric emptying in the SADJB-SG rats before sacrifice. **c** Representative images of both experimental groups, before surgery and sacrifice. Linear mixed models were used for repeated measures analysis over time. Estimated *mean* ± *SE* was represented, and Bonferroni method was used to calculate adjusted *p*-values: *p < 0.05 and **p < 0.001

There was no difference in stomach area in the pre-operative radiological study. Values were around 5 cm^2^, both in Sham and SADJB-SG animals. Before sacrifice, the radiological study showed that the mean area of the stomach did not change in the Sham group compared to pre-surgery values, whereas in SADJB-SG, it was 42% smaller than the stomach in the Sham group (Fig. 5a). Indeed, gastric emptying was accelerated in SADJB-SG rats, and the effect was significant between 6 and 8 h after barium administration (Fig. 5b); moreover, it was even evident in the radiological images at *t* = 0, where a significant amount of barium could be seen in the intestine of the operated rats (Fig. 5c).

### Histological Evaluation of the Esophagus

**Fig. 6:**
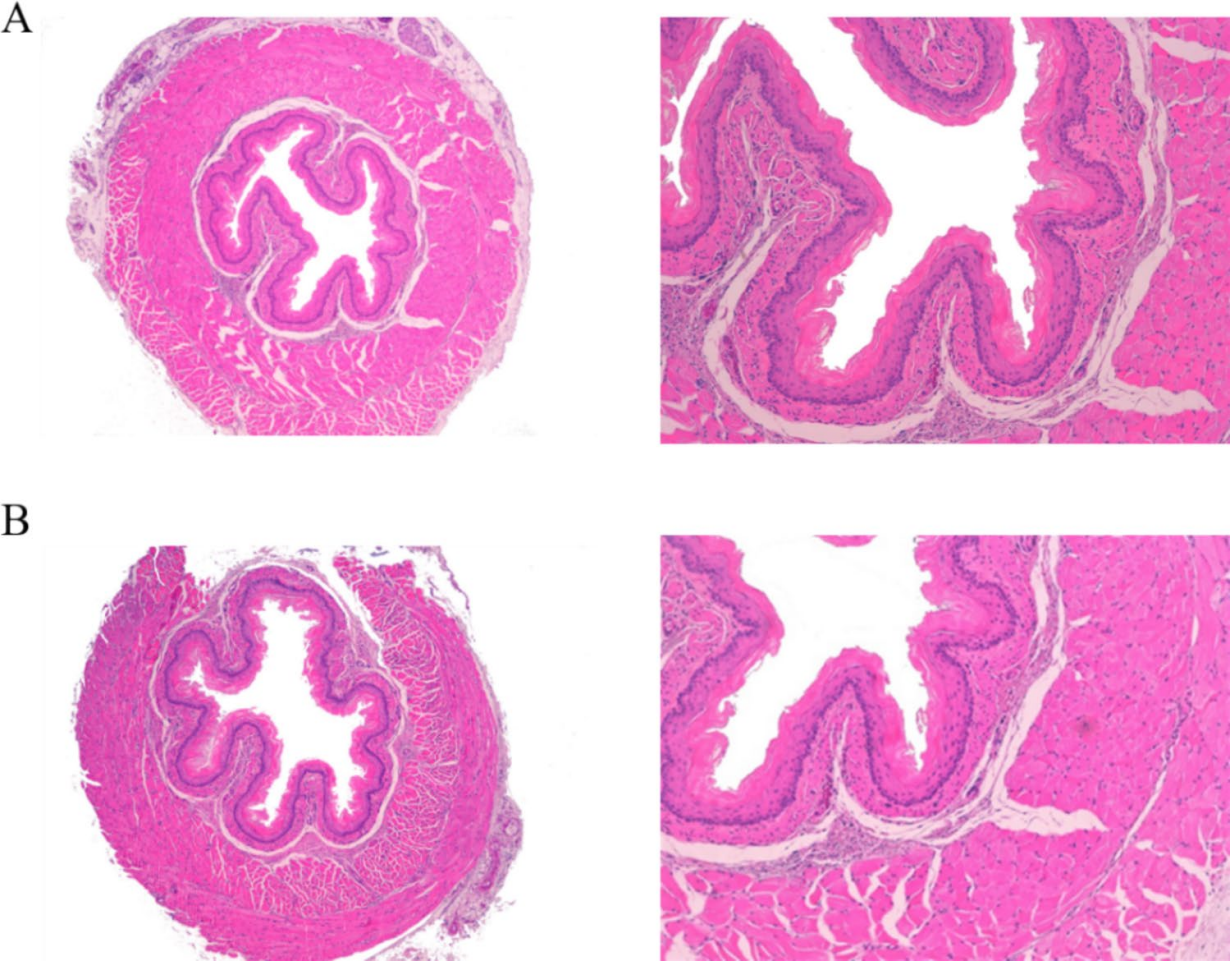
Representative light microphotographs corresponding to hematoxylin–eosin staining in the Sham (**a**) and in the SADJB-SG (**b**) groups. Magnification: 40 × , on the left; 100 × , on the right

Distal esophagus histology was normal in both SADJB-SG and Sham groups. Specifically, no signs of esophagitis such as mucosal and submucosal eosinophilia were detected in any experimental group (Fig. 6).

## Discussion

Despite promising pharmacotherapies, metabolic surgery remains the most effective strategy for the treatment of obesity and T2DM [22, 23]. In this study, SADJB-SG was performed in GK rats with similar mortality and associated medical complications, as previously described [16]. We observed that SADJB-SG led to substantial weight loss despite similar food intake compared to the Sham group from the second week post-surgery. These results are similar to those reported both in humans and GK rats [22, 24]. However, weight loss was higher than we expected, as final stomach size reduction in our surgical modification was 42% measured on the radiographic study, instead of the usual 80% resection in sleeve gastrectomy [24–26]. According to the analyses we have carried out in this study, we are unable to determine whether this weight loss is due to a gut rerouting or to a reduction in energy intake as described in other metabolic surgeries in humans [27].

On the other hand, coupled with weight loss, SADJB-SG led to a decrease in serum albumin levels that could indicate a poorer nutritional status in SADJB-SG group. Protein deficiency after metabolic bariatric surgery remains the most severe macronutrient complication in bariatric surgery, especially in derivative procedures such as BPD and RYGB, where protein supplementation might be needed [23].

An improvement in glucose homeostasis following SADJB-SG was observed. Fasting glycemia and HOMA-IR showed a significant long-term decrease after surgery, indicating a lower insulin resistance in these animals [12, 28]. Furthermore, elevated fasting insulin levels were found in the SADJB-SG group. This finding could be correlated with improved pancreatic beta-cell function as a consequence of the surgical procedure [26, 29, 30]. Moreover, fructosamine was significantly lower in the SADJB-SG group at follow-up supporting the improvement in glucose homeostasis during the preceding 2 weeks before sacrifice [31].

However, post-surgery and follow-up OGTT tests showed a significant increase in glycemia 30 min after overload. There is evidence to support this phenomenon that has also been observed after vertical sleeve gastrectomy (VSG) in mice [32] or following RYGB [30] and sleeve gastrectomy in GK rats [26]. This increase implied a higher post-surgical *AUC* in the SADJB-SG animals. *AUC* is an index of whole glucose excursion after glucose loading that reflects glycemic variability, which is proposed to be harmful for blood vessels and affect the development of medical complications [19]. In our model, we can attribute this increase in glycemia after overloading to a faster gastric emptying as observed in previous studies, performing VSG and RYGB [33]. This phenomenon could increase glucose availability in the jejunum, where anastomosis was performed in SADJB-SG, and most of the glucose uptake occurs through the sodium-dependent glucose transporter 1 (SGLT1) [34].

From a clinical point of view, an important issue related to sleeve and sleeve-plus procedures is the development of new-onset gastroesophageal reflux disease (GERD), or even a worsening of GERD symptoms prior to surgery [35]. A meta-analysis showed a significant worsening of GERD in 19% of the patients and 23% of de novo GERD [36]. Moreover, Ser and colleagues reported a 5-year follow-up study demonstrating de novo GERD in 30.6% of patients after SADJB-SG [15]. GERD has also been described in rats after sleeve gastrectomy [37]. Despite GERD was not demonstrated in either the SADJB-SG or the SHAM group in our study, this procedure cannot be recommended in all types of patients on the sole basis of this finding.

This study has some limitations. Data concerning other hormones involved in glucose metabolism (i.e., ghrelin, GLP-1, GIP…) would be needed to fully understand the metabolic effects of SADJB-SG in GK rats. On the other hand, the translation of results obtained in animal models to humans is complex and the risks/benefits balance must be carefully evaluated [15, 38].

## Conclusion

SADJB-SG technique in GK rats led to an improvement in glucose homeostasis. Further studies are needed to elucidate the molecular mechanisms involved in insulin resistance and glucose metabolism improvement.

## Data Availability

No datasets were generated or analysed during the current study.

## Abbreviations

BDP: Biliopancreatic diversion
DS: Duodenal switch
GERD: Gastroesophageal reflux disease
GK: Goto-Kakizaki
HOMA-IR: Homeostasis model assessment of insulin resistance
OGTT: Oral glucose tolerance test
RYGB: Roux-en-Y gastric bypass
SADI-S: Single anastomosis duodenoileal bypass with sleeve gastrectomy
SADJB-SG: Single anastomosis duodenojejunal bypass with sleeve gastrectomy
T2DM: Type 2 diabetes mellitus
